# The causality between intestinal flora and allergic diseases: Insights from a bi-directional two-sample Mendelian randomization analysis

**DOI:** 10.3389/fimmu.2023.1121273

**Published:** 2023-03-09

**Authors:** Qiubai Jin, Feihong Ren, Dan Dai, Nan Sun, Yiyun Qian, Ping Song

**Affiliations:** ^1^ Guang’anmen Hospital, China Academy of Chinese Medical Sciences, Beijing, China; ^2^ Graduate School, Beijing University of Chinese Medicine, Beijing, China; ^3^ Peking Union Medical College Hospital, Chinese Academy of Medical Science, Beijing, China

**Keywords:** Mendelian randomization, intestinal flora, atopic dermatitis, allergic rhinitis, allergic asthma, causality

## Abstract

**Background:**

Growing evidence shows a significant association between intestinal flora and allergic diseases, specifically atopic dermatitis (AD), allergic rhinitis (AR), and allergic asthma (AA). However, the causality has not yet been clarified.

**Objective:**

We conducted a bidirectional two-sample Mendelian randomization (TSMR) analysis to study the causal relationships between intestinal flora classification and AD, AR, or AA.

**Materials and methods:**

We obtained summary data of intestinal flora, AD, AR, and AA from a genome-wide association research. The inverse-variance weighted method is the primary method for analyzing causality in the TSMR analysis. Several sensitivity analyses were conducted to examine the stability of TSMR results. Reverse TSMR analysis was also performed to assess whether there was a reverse causality.

**Results:**

A total of 7 bacterial taxa associated with AD, AR, and AA were identified by the current TSMR analysis. Specifically, the genus Dialister(*P*=0.034)and genus Prevotella(*P*=0.047)were associated with a higher risk of AD, whereas class Coriobacteriia (*P*=0.034) and its child taxon, order Coriobacteriales (*P*=0.034) and family Coriobacteriaceae (*P*=0.034), all had a protective effect on AR. In addition, the family Victivallaceae (*P*=0.019) was identified as a risk factor for AR. We also noticed a positive association between the genus Holdemanella (*P*=0.046) and AA. The reverse TSMR analysis didn’t suggest any evidence of reverse causality from allergic diseases to the intestinal flora.

**Conclusion:**

We confirmed the causal relationship between intestinal flora and allergic diseases and provided an innovative perspective for research on allergic diseases: targeted regulation of dysregulation of specific bacterial taxa to prevent and treat AD, AR, and AA.

## 1 Introduction

The incidence of atopic dermatitis (AD), allergic rhinitis (AR), and allergic asthma (AA) have dramatically increased over the past three decades, resulting in a considerable burden to society (1). AD is a common inflammatory skin disease characterized by recurrent eczematous lesions. AR is a non-infectious inflammatory disease of the nasal mucosa, with typical symptoms of paroxysmal sneezing, watery nasal discharge, nasal itching and congestion. AA is an airway inflammatory disease that results in recurring wheezing, chest tightness, shortness of breath, and mucus production. Currently, there is no effective radical treatment for AD, AR, or AA. Glucocorticoids and antihistamines are commonly used for treating these three allergic diseases; but symptoms tend to rebound after drug withdrawal (2). AD, AR and AA are associated with genetic, dietary, and environmental factors (such as air pollution and exposure to allergens); however, the underlying causes remain unclear (3). Therefore, there is an urgent need to identify potential causal risk factors for AD, AR and AA.

The intestinal flora is a dynamic ecosystem known as the ‘second genome’ (4). Intestinal dysbiosis can cause metabolic disorders of intestinal microorganisms and further lead to immune dysfunction (5). With an in-depth study of the gut-skin and gut-lung axes, more attention has been paid to the effect of intestinal flora on the skin and respiratory tract (6–8). Several observational studies have reported that the abundance of certain intestinal flora changes significantly in patients with AD, AR, and AA compared to healthy individuals, indicating a potential correlation between intestinal flora and these three allergic diseases (9–11). Intestinal flora can regulate adaptive immunity by maintaining the balance between effector T cells (Th1, Th2, and Th17) and regulatory T cells, which may explain the effect of intestinal flora on AD, AR and AA (12, 13).

However, owing to the lack of evidence from randomized controlled trials, it is unclear whether there is a definite causality between intestinal flora and these three allergic diseases. As the gold standard for causal inference in epidemiological studies, randomized controlled trials are sometimes difficult to conduct because of ethical limitations and high costs. Two sample Mendelian randomization (TSMR) is an effective alternative (14). Genome-wide association studies (GWAS) have made great contributions to the identification of genetic variants related to diseases, mainly single nucleotide polymorphism (SNP), which can increase our understanding of the genetic basis of many complex traits in human diseases (15). TSMR uses genetic variation related to exposure as an alternative indicator of exposure to study the causality between exposure and outcome (16). The selected SNPs are also called instrumental variances (IVs). TSMR simulates randomization based on the random distribution of genetic variants during gametogenesis, conceptually similar to randomized controlled trials (17). Since these genetic variants precede diseases progression and are independent of lifestyle and environmental factors after birth, TSMR can minimize the influence of confounding factors and reverse causality (16).

In this study, we used the latest available GWAS database published in 2021 (18) for TSMR analysis to investigate the possible causality between intestinal flora and AD, AR, and AA, to provide an innovative perspective for the research of allergic diseases: targeted regulation of specific bacterial taxa to prevent and treat AD, AR, and AA.

## 2 Materials and methods

### 2.1 Study design

**Figure 1:**
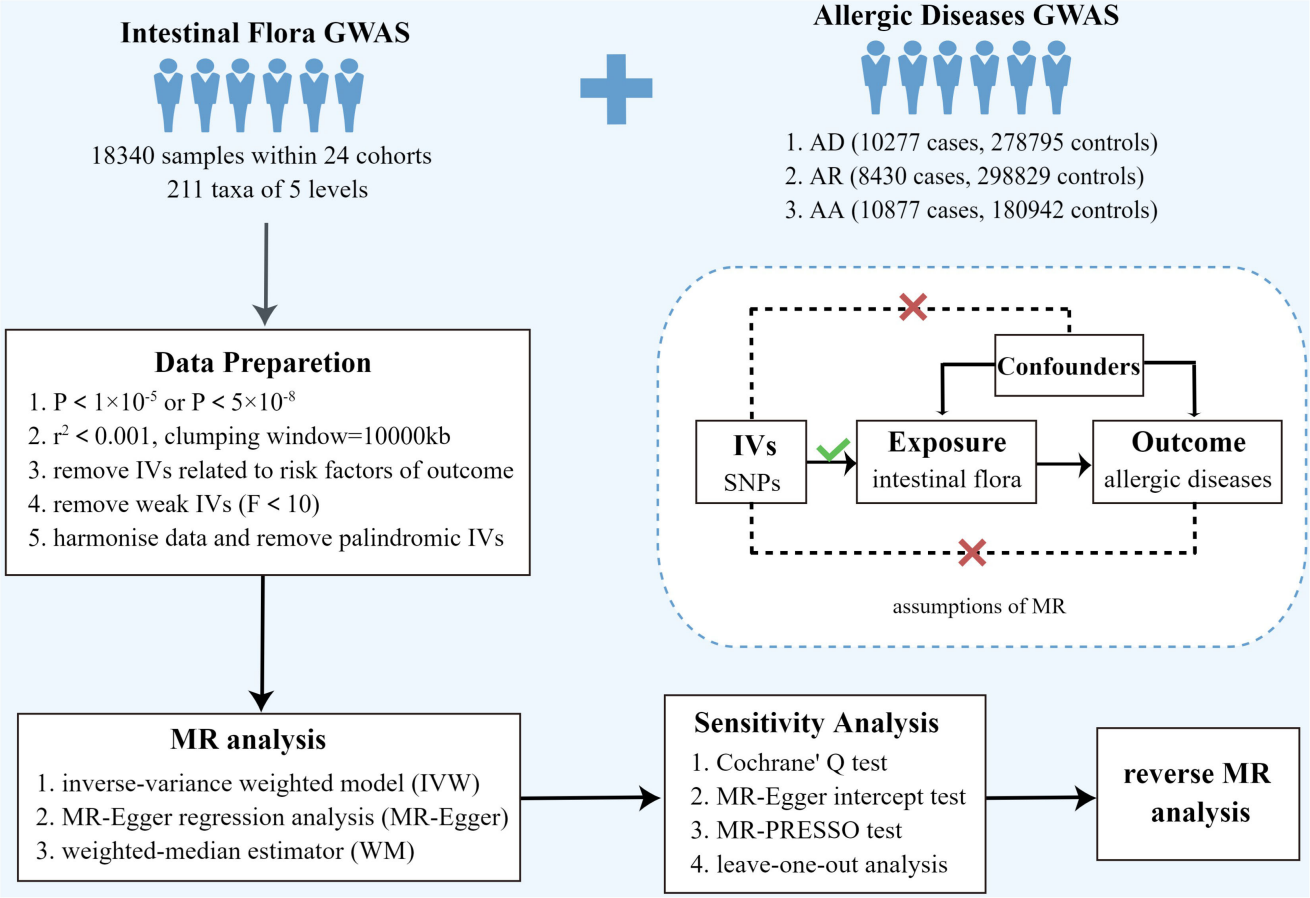
Overview of present MR analyses and assumptions. AD, atopic dermatitis; AR, allergic rhinitis; AA, allergic asthma; SNP, single nucleotide polymorphism.

TSMR was used to analyze the causal relationship between intestinal bacterial taxa and allergic diseases (AD, AR, and AA). The overall design of this study is shown in **Figure 1**. To obtain reliable results, three hypotheses need to be satisfied when performing TSMR analysis (1): there is a strong correlation between genetic variants and exposure factors (2); there is no correlation between genetic variants and confounders; and (3) genetic variants can only affect the outcome through exposure factors, but not through other methods, that is, horizontal pleiotropy is not allowed (**Figure 1**). Genetic variants that satisfy these three hypotheses can be included in TSMR analysis as instrumental variables (16).

### 2.2 Data sources and selection of instrumental variables

Summary statistics of the intestinal flora were obtained from a large-scale GWAS study by the MiBioGen consortium (https://mibiogen.gcc.rug.nl), involving 18340 European ethnic participants from 11 countries with 122,110 loci of variation (18). We screened the IVs of intestinal bacterial taxa at five levels (phylum, class, order, family, and genus) from this GWAS. Fifteen bacterial taxa without specific species names were eliminated. The GWAS statistics for AD, AR, and AA were obtained from the data released by FinnGen Research (https://r7.finngen.fi/) in July 2022. The diagnostic criteria of AD were based on ICD-8, ICD-9 and ICD-10 standards and the GWAS statistics contain 16,383,295 loci of variations from 10,277 cases and 278,795 controls. The diagnostic criteria of AR were based on ICD-9, and ICD-10 standards, and the GWAS statistics of AR contain 16383313 loci of variation from 8430 cases and 298829 controls. The diagnostic criteria of AA were based on ICD-10 standards and the GWAS statistics of AR contain 16383313 loci of variation from 8430 cases and 298829 controls.

To obtain more complete results, we used a genome-wide significance threshold (5 × 10^-8^) and a locus-wide significance threshold (1 × 10^-5^), respectively to screen SNPs related to exposure (19, 20). Linkage disequilibrium analysis was performed to satisfy TSMR’s hypothesis 1. The linkage disequilibrium correlation coefficient was set to r^2^<0.001 and clumping window >10000kb to ensure no linkage disequilibrium among the included IVs. To avoid horizontal pleiotropy, IVs associated with risk factors for allergic diseases were excluded using PhenoScanner V2 (21). Palindromic and incompatible SNPs were excluded when harmonizing the statistics of exposure and outcome, and SNPs related to exposure that could not be matched in the GWAS outcome statistics were excluded. To avoid the influence of weak instrument bias on causal inference, we used the formula F=β^2^_exposure_/SE^2^_exposure_ to calculate the strength of the IVs (16, 20, 22) and eliminate IVs with F < 10 (23).

### 2.3 Statistical analysis

TSMR was conducted to analyse the causality between bacterial taxa and AD, AR, and AA. In the absence of horizontal pleiotropy, the inverse-variance weighted (IVW) method can be the primary method for analyzing causality in TSMR analysis (24). Before that, we implemented the Cochrane’s Q test to evaluate the heterogeneity between the IVs. If heterogeneity was detected(*P*<0.05), the random-effects IVW model could provide a more conservative estimate; otherwise, the fixed-effect IVW model would be used (25). Other methods of TSMR analysis, including the weighted median estimator (WM) and MR-Egger regression (26), can supplement the IVW method and provide wider confidence intervals (27). These three TSMR methods for causal inference have their model assumptions. The IVW method is suitable for situations where horizontal pleiotropy does not exist (24); the WM method assumes that less than 50% of IVs have horizontal pleiotropy (28). The MR-Egger regression assumes that more than 50% of IVs are affected by horizontal pleiotropy (26).

If the result of the TSMR analysis was nominally significant (*P* < 0.05), we considered that there might be a causal relationship between the flora and outcome (29). If the causality between bacterial taxa and outcome is identified as significant by two or more TSMR methods, the result is considered robust (5).

The existence of horizontal pleiotropy may challenge the second TSMR hypothesis; therefore, we adopted various methods to monitor possible horizontal pleiotropy. Specifically, the p-value of the MR-Egger intercept test and MR pleiotropy residual sum and outlier (MR-PRESSO) global test can be used to assess the existence of horizontal pleiotropy, and *P* < 0.05 was considered statistically significant (5, 30). The MR-PRESSO outlier test can adjust horizontal pleiotropy by detecting and removing outliers (31), and the number of distributions in the MR-PRESSO analysis was set to 1000 (17).

Additionally, we conducted a leave-one-out sensitivity analysis of the identified significant results to determine whether the causal relationship of the TSMR analysis was caused by a single SNP (32). Finally, a reverse TSMR analysis was performed between allergic diseases (AD, AR and AA) and the identified significant bacterial taxa using positive TSMR analysis to examine whether a reverse causal association existed. The reverse TSMR procedure was the same as that for the above TSMR analysis. TSMR analyses were performed using the ‘TwoSampleMR’ (version 0.5.6) in R software (version 4.2.1).

### 2.4 Ethical approval

The GWAS data used in this study were public de-identified data. The ethics committee approved these data; therefore, there was no need for additional ethical approval.

## 3 Results

IVs were screened according to the conditions described above. The details of the SNPs, that were eventually included in the TSMR analysis of intestinal flora and allergic diseases, are presented in **Supplementary Table 1**. After harmonization, the number of SNP involved in each pair of bacterial taxa and allergic diseases was more than three. The F-statistics of all SNPs were greater than ten, indicating that there are no weak IVs. Moreover, it should be noted that there is an inclusive relationship between intestinal flora classifications. Thus, the SNPs included in the class and their relevant order may overlap heavily. For example, SNPs of the order Coriobacteriales, class Coriobacteriia, and family Coriobacteriaceae.

### 3.1 Results of the TSMR analysis (locus-wide significance, *P*<1×10^-5^)

**Figure 2:**
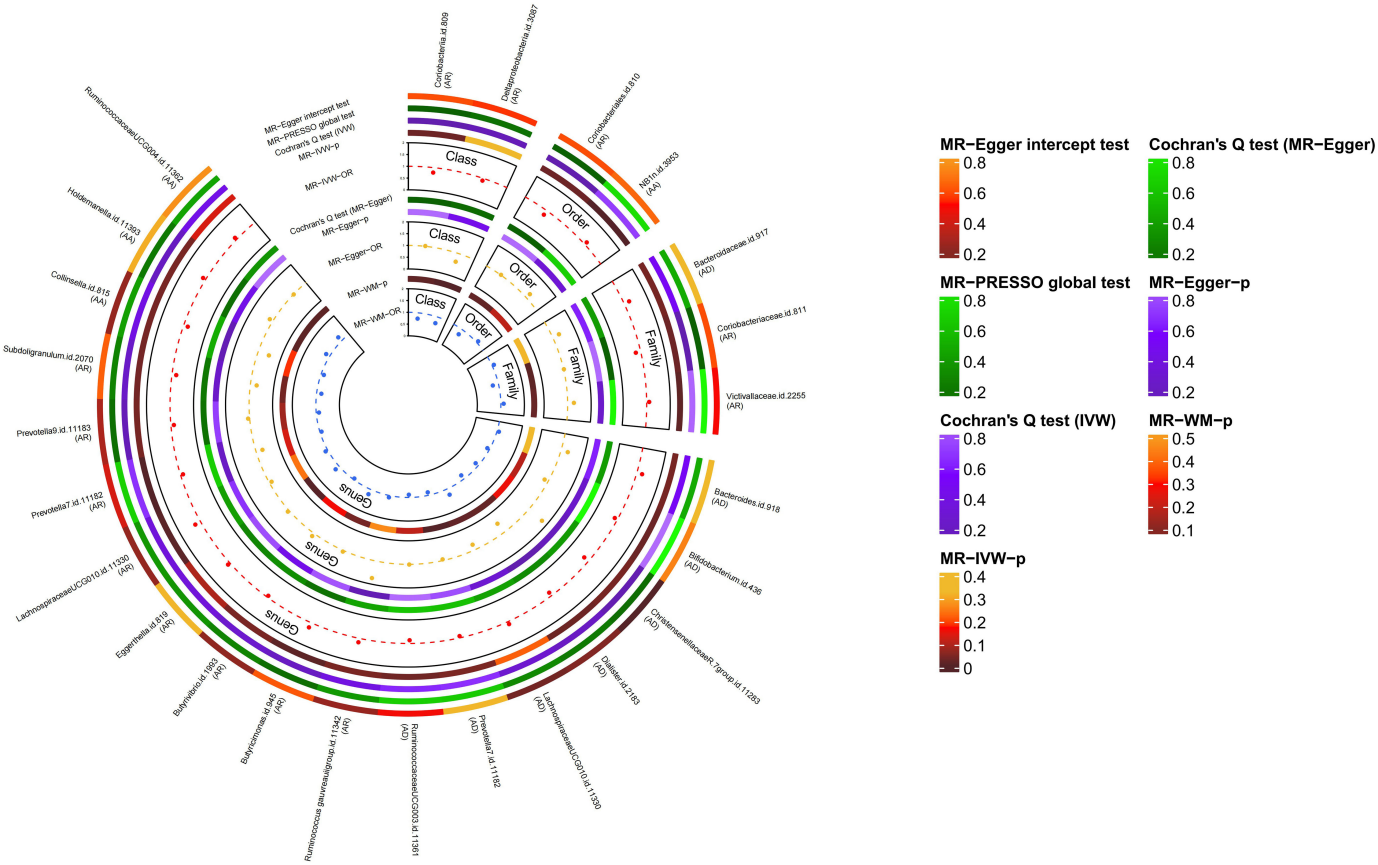
Causal analysis of intestinal flora and allergic diseases (locus-wide significance, P<1×10^-5^). MR-PRESSO, Mendelian Randomization Pleiotropy Residual Sum and Outlier; IVW, inverse-variance weighted method; WM, weighted median estimator; AD, atopic dermatitis; AR, allergic rhinitis; AA, allergic asthma.

**Figure 3:**
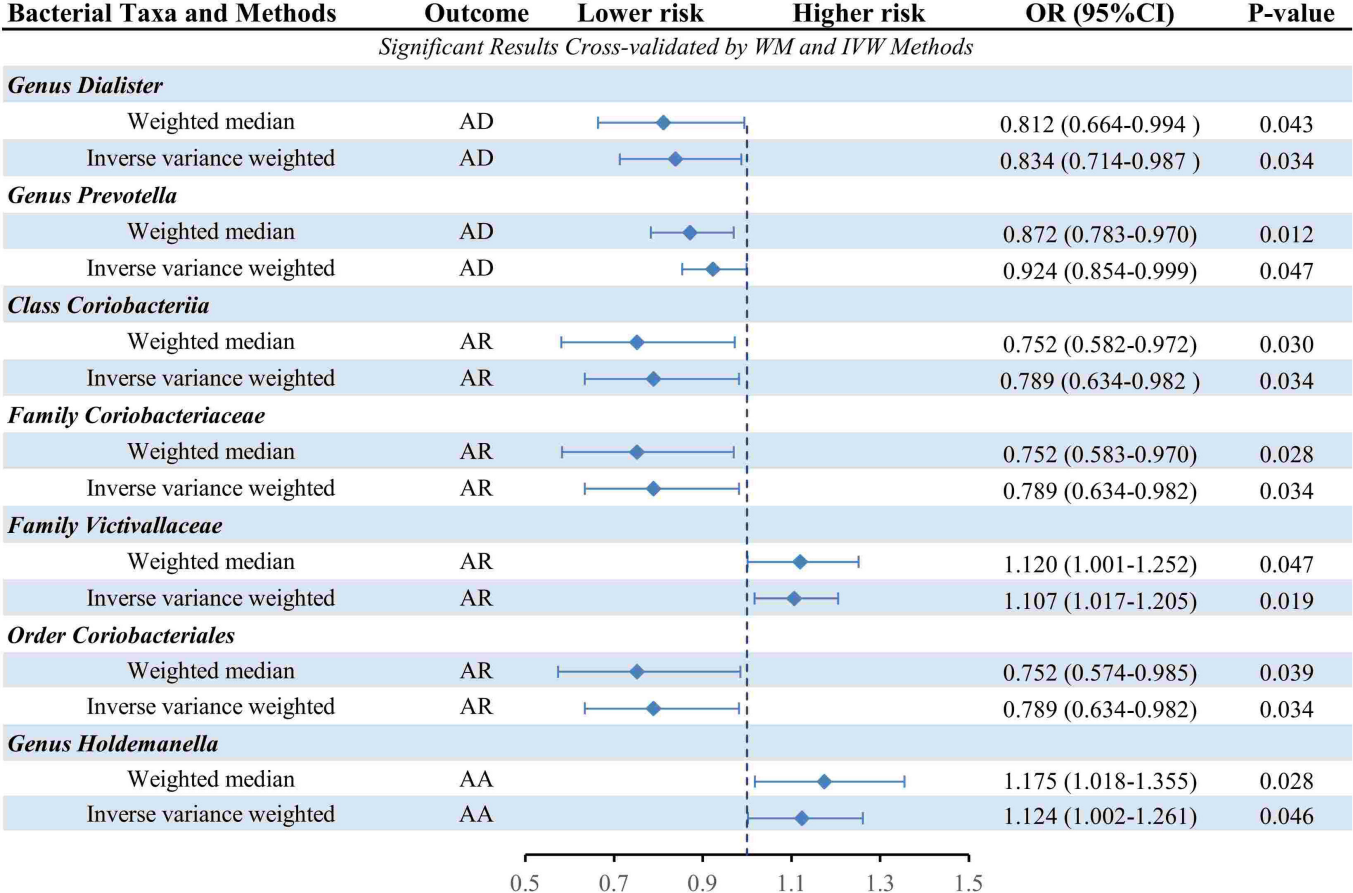
Forest plot of the causality between cross-validated 7 bacterial taxa with the risks of AD, AR, or AA. IVW, inverse-variance weighted method; WM, weighted median estimator; AD, atopic dermatitis; AR, allergic rhinitis; AA, allergic asthma.

The causal relationship between each pair of bacterial taxa and allergic disease was analyzed using the three TSMR methods (**Supplementary Table 2**). Twenty-five potential causal associations between bacterial traits and allergic diseases were identified using one or more TSMR methods (**Figure 2**). Among them, two bacterial taxa related to AD, four bacterial taxa associated with AR and one bacterial taxon related to AA were cross-validated using the IVW and WM methods (**Table 1** and **Figure 3**). We mainly focused on these seven relatively stable causal relationships.

**Table 1:** Summary of causality between intestinal flora and AD, AR or AA (*P*<1×10^-5^).

Human gut microbiota	Nsnps	Traits	Method	OR	95%CI	*P*-value
*Genus Dialister*	11	AD	WM	0.812	0.664-0.994	0.043
			IVW	0.834	0.714-0.987	0.034
*Genus Prevotella*	11	AD	WM	0.872	0.783-0.970	0.012
			IVW	0.924	0.854-0.999	0.047
*Class Coriobacteriia*	13	AD	WM	0.752	0.582-0.972	0.030
			IVW	0.789	0.634-0.982	0.034
*Family Coriobacteriaceae*	13	AR	WM	0.752	0.583-0.970	0.028
			IVW	0.789	0.634-0.982	0.034
*Family Victivallaceae*	11	AR	WM	1.120	1.001-1.252	0.047
			IVW	1.107	1.017-1.205	0.019
*Order Coriobacteriales*	13	AR	WM	0.752	0.574-0.985	0.039
			IVW	0.789	0.634-0.982	0.034
*Genus Holdemanella*	11	AA	WM	1.175	1.018-1.355	0.028
			IVW	1.124	1.002-1.261	0.046

AD, atopic dermatitis; AR, allergic rhinitis; AA, allergic asthma; Nsnp, number of single nucleotide polymorphism; WM, weighted median estimator; IVW, inverse-variance weighted method; OR, odd ratio; CI, confidence interval.

We also performed the leave-one-out sensitivity analysis for the identified significant bacterial taxa, and the results further validated the robustness of our results (**Supplement Figure 1**). In the absence of heterogeneity, horizontal pleiotropy, and outliers, the results of TSMR analysis are credible.

#### 3.1.1 AD

Seven causal associations from bacterial taxa to AD were identified by the IVW method. Considering the cross-validation, the results of the two bacterial taxa remained stable. In specific, our TSMR analysis found that genus Dialister (OR: 0.839, 95% confidence interval (CI): 0.714-0.987, *P*=0.034) and genus Prevotella (OR: 0.924, 95% CI: 0.854-0.999, *P*=0.047) were associated with a higher risk of AD. In the sensitivity analysis, Cochrane’s Q test did not suggest evidence of heterogeneity in the genus Dialister (*P*=0.214) and genus Prevotella (*P*=0.672) (**Supplementary Table 3**). The MR-Egger intercept test observed no horizontal pleiotropy in the genus Dialister (*P*=0.188) and genus Prevotella (*P*=0.914) (**Supplementary Table 4**). Similarly, MR-PRESSO global test didn’t detect any horizontal pleiotropy in the genus Dialister (*P*=0.231) and genus Prevotella (*P*=0.667) (**Supplementary Table 5**). For the MR-PRESSO outlier test, no outlier was found in the genus Dialister and genus Prevotella (**Supplementary Table 5**).

#### 3.1.2 AR

Eleven causal relationships from bacterial taxa to AR were identified by the IVW method. Considering the cross-validation, the results of the four bacterial taxa remained stable. Specifically, class Coriobacteriia (OR: 0.789, 95% CI: 0.634-0.982, *P*=0.034) and its child taxon, order Coriobacteriales and family Coriobacteriaceae, all had a protective effect on AR. On the contrary, the family Victivallaceae (OR: 1.107, 95% CI: 1.017-1.205, *P*=0.019) was associated with a higher risk of AR. In the sensitivity analysis, Cochrane’s Q test did not suggest evidence of heterogeneity in class Coriobacteriia (*P*=0.071), family Coriobacteriaceae (*P*=0.071), family Victivallaceae(*P*=0.938) and order Coriobacteriales(*P*=0.071) (**Supplementary Table 3**). No horizontal pleiotropy was observed by MR-Egger intercept test in class Coriobacteriia (*P*=0.609), family Coriobacteriaceae (*P*=0.609), family Victivallaceae(*P*=0.507) and order Coriobacteriales(*P*=0.609) (**Supplementary Table 4**). Similarly, MR-PRESSO global test didn’t detect any horizontal pleiotropy in class Coriobacteriia (*P*=0.081), family Coriobacteriaceae (*P*=0.081), family Victivallaceae(*P*=0.938) and order Coriobacteriales(*P*=0.949) (**Supplementary Table 5**). For the MR-PRESSO outlier test, no outlier was found in class Coriobacteriia, family Coriobacteriaceae, family Victivallaceae and order Coriobacteriales(**Supplementary Table 5**).

#### 3.1.3 AA

Three causal associations from bacterial taxa to AA were identified by the IVW method. Considering the cross-validation, only one bacterial taxon remained stable. Specifically, our TSMR analysis found that genus Holdemanella (OR: 1.124, 95% CI: 1.002-1.261, *P*=0.046). In the sensitivity analysis, Cochrane’s Q test did not suggest evidence of heterogeneity in the genus Holdemanella (*P*=0.198) (**Supplementary Table 3**). No horizontal pleiotropy was observed by the MR-Egger intercept test in the genus Holdemanella (*P*=0.886) (**Supplementary Table 4**). Similarly, MR-PRESSO global test didn’t detect any horizontal pleiotropy in the genus Holdemanella (*P*=0.227) (**Supplementary Table 5**). For the MR-PRESSO outlier test, no outlier was found in the genus Holdemanella(**Supplementary Table 5**).

### 3.2 Results of the TSMR analysis (genome-wide significance threshold, *P*<5 × 10^-8^)

In the TSMR analysis of intestinal flora as a whole and allergic diseases, the IVW, WM, and MR-Egger regression methods did not find any significant causal associations. In the sensitivity analysis, Cochrane’s Q test did not suggest evidence of heterogeneity, MR-Egger intercept test and MR-PRESSO global test didn’t detect any horizontal pleiotropy, and the MR-PRESSO outlier test didn’t find any outliers. All the results are shown in **Supplementary Table 6**.

### 3.3 Reverse TSMR analysis

The results of reverse TSMR analysis are presented in **Supplementary Table 7**. Considering cross-validation, we did not find any reverse causal relationships between the intestinal flora classification shown in **Table 1** and allergic diseases.

## 4 Discussion

**Figure 4:**
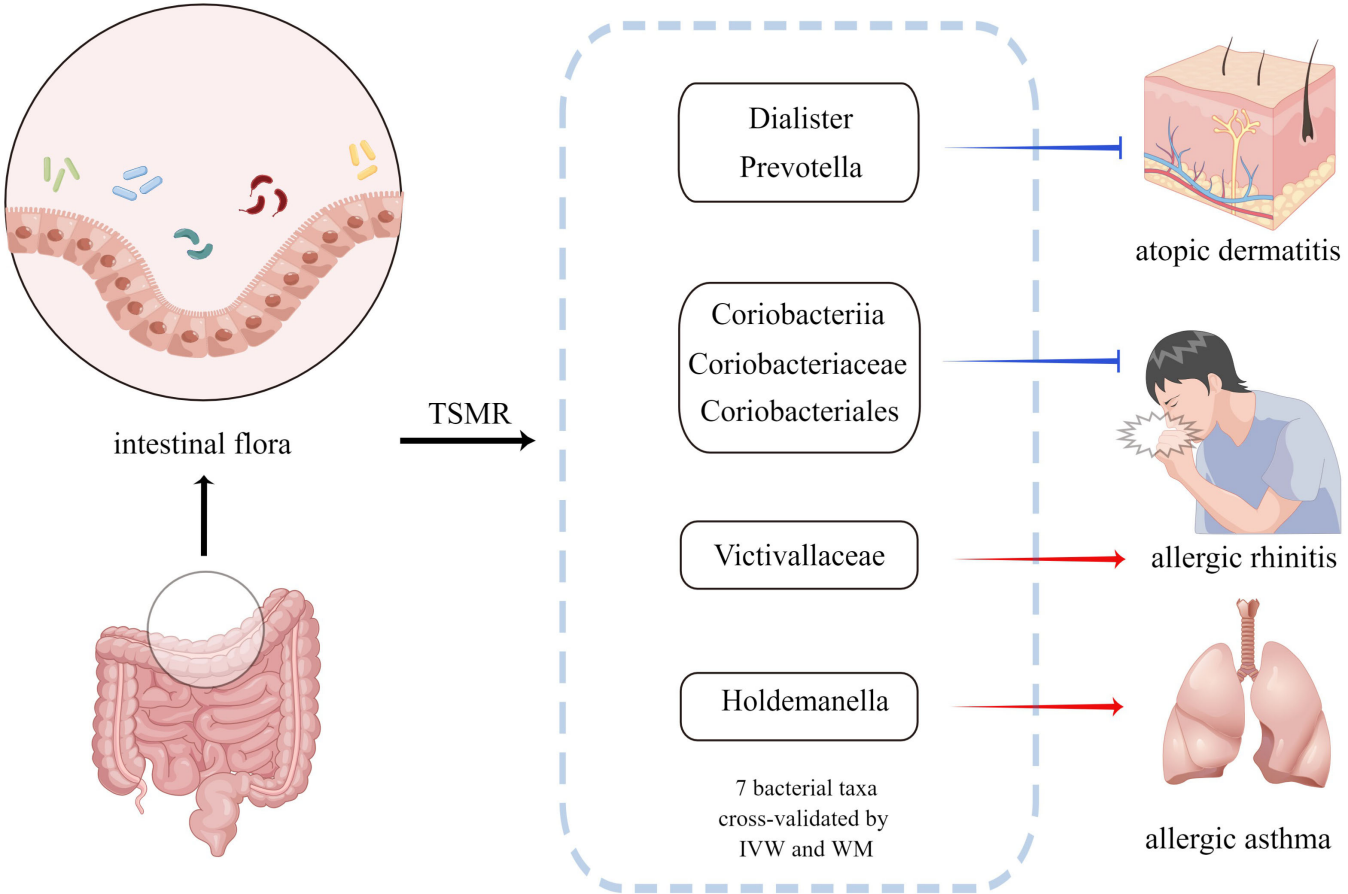
Bacterial taxa associated with AD, AR or AA identified by the current MR analysis. The blue arrow indicates that the bacterial taxa is the protective factor of the outcome and the red arrow indicates that the bacterial taxa is the risk factor of the outcome. MR, Mendelian randomization; IVW, inverse-variance weighted method; WM, weighted median estimator.

Using large-scale GWAS statistics, seven bacterial traits associated with AD, AR, and AA were identified by the current TSMR analysis (**Figure 4**). In accordance with some prospective observational studies (10, 33) and animal experiments (34, 35), our TSMR study also revealed that the genus Dialister and genus Prevotella may be protective factors for AD. Th2-skewed and Th17-skewed immune dysregulation are some of AD’s most significant pathogenesis mechanisms (36), whereas Treg cells can inhibit Th2 and Th17 allergic inflammation and restore immune tolerance (37). Furthermore, the genus Dialister is a propionate producer in the intestinal tract (38), and genus Prevotella can break down fibers and produce propionate and butyrate (39). Several studies have discovered that the content of short-chain fatty acids (SCFAs) such as propionate and butyrate in patients with AD is significantly lower than that in healthy individuals (40, 41). As important SCFAs, propionate and butyrate can inhibit the Th2-skewed and Th17-skewed inflammation in AD. Specifically, both can inhibit histone deacetylase (42) and induce the differentiation of peripheral CD4+T cells to Treg cells, thus producing anti-inflammatory cytokine IL-10 and inhibiting the function of Th2 and Th17 cells (43). In addition, some studies have shown that butyrate can inhibit the release of histamine and other inflammatory mediators from mast cells by inhibiting the interaction between Immunoglobulin E and mast cells (44). Therefore, we speculate that the protective effect of these two bacterial taxa on AD might be related to SCFAs, especially propionate and butyrate.

The Genus Holdemanella was considered as risk factor for AA based on the TSMR analysis. Reportedly, the abundance of the genus Holdemanella is negatively correlated with the propionate content in patients with diabetes and cognitive impairment (45). Thus, we speculate that the genus Holdemanella may promote Th2 inflammation in AA by affecting SCFAs such as propionate (46). However, the exact mechanism is still unclear and it is necessary to further study the possible role of the genus Holdemanella.

Class Coriobacteriia and its child taxa, order Coriobacteriales, and family Coriobacteriaceae, all have negative effects on AR, whereas, family Victivallaceae is the risk factor for AR. The relationship between these intestinal florae and allergic diseases was cross-validated using two IVW and WM methods. However, the function of this bacteria is poorly understood. Currently, there are no studies on the relationship between these bacterial taxa and allergic diseases, and the specific flora of AR and AA were reported for the first time in this study. Therefore, our study may provide a new perspective for mechanistic research on AR and AA.

In addition to the seven stable causal associations above, the IVW method yielded several interesting results, which are supported by previous studies. We also discovered that the family Bacteroidaceae and its child taxon genus Bacteroides were both risk factors for AD. Studies have reported that the abundance of the genus Bacteroides in patients with AD is significantly higher than that in healthy people (47), and its proportion is positively correlated with the severity of AD symptoms (33). Lipopolysaccharide, the metabolite of the family Bacteroidaceae and genus Bacteroides, can promote Th2 inflammation in AD (48). Moreover, the genus Bacteroides and genus Prevotella share a common ancestor but have inhibitory effects on each other (49). Genus Bacteroides is dominant in people who consume protein and animal fat in their main diet. In contrast, genus Prevotella is predominant in people who take fruits and vegetables as their main diet (50). The research of Nosrati et al. showed that vegetable intake could improve AD symptoms (51). This suggests that the relationship between intestinal flora and AD can be studied from the perspective of diet in the future. After all, it is much easier to adjust eating habits than to change genetic or environmental factors.

Butyrate, a metabolite of gut microbiota, may be an important connector between gut microbiota and allergic diseases (52). Genus Subdoligranulum (53) and genus Collinsella (54), both butyrate producers, were also determined to be protective factors of AR and AA by the IVW method. However, there are some differences in the role of butyrate in patients with AR and AA. Specifically, Th2-skewed and Th17-skewed immune dysregulation are important pathogenic mechanisms of AR (55). Similarly, the genus Subdoligranulum may also inhibit Th2 and Th17-mediated inflammation in AR by producing butyrate. In addition, IL-4, an important cytokine in Th2 inflammation, can impair the airway epithelial barrier function in AR patients. In this case, butyrate can improve the function of the airway epithelial barrier (56), which may explain the protective effect of genus Subdoligranulum on AR. Moreover, the inflow of eosinophils into the lung parenchyma is a hallmark of AA (46). Butyrate flows into eosinophils through monocarboxylate transporters and promotes apoptosis (57). In addition, type 2 innate lymphoid cells (ILC2) can promote T2 immunity in AA (46). Butyrate can inhibit the release of type 2 immune factors such as IL-5 and IL-13 by ILC2 cells, which can reduce the inflammatory response of AA (58).

This study has several advantages. Firstly, this is the first bi-directional TSMR study to reveal the causal relationship between intestinal flora and allergic diseases (AD, AR, and AA), which is not disturbed by confounding factors or reverse causality. Second, we set strict conditions for the screening of instrumental variables, and only when more than two TSMR methods identify the causal relationship can it be considered conceivable. Thirdly, we provided evidence for research on the intestinal-skin and intestinal-lung axes from a genetic perspective. Seven bacterial taxa associated with AD, AR, and AA were identified using TSMR analysis. These identified significant bacterial taxa could serve as candidate microbiome interventions in future clinical trials of allergic diseases. Meanwhile, our findings may provide an innovative perspective for research on allergic diseases: targeted regulation of specific bacterial taxa such as supplementing beneficial bacteria and inhibiting the growth of harmful bacteria to prevent and treat AD, AR and AA.

There are also some limitations of this study. Firstly, the number of instrumental variables involved in GWAS statistics of intestinal flora is limited, and there are no data available at the species level. Secondly, we could not determine whether there were overlapping participants in the GWAS data of the exposures and outcomes involved in this study. Thirdly, demographic data were lacking in the original research; therefore, we could not perform subgroup analysis on factors such as gender. Our results need to be verified by further clinical and basic research. In future study, we will increase the sample size and more accurately explore the relationship between intestinal flora and allergic diseases at the species level.

## Data availability statement

The original contributions presented in the study are included in the article/**Supplementary Material**. Further inquiries can be directed to the corresponding author.

## Author contributions

PS and QJ designed the study. NS and YQ collected the data. QJ, FR, DD, and NS performed the computations and manuscript writing. All authors contributed to the article and approved the submitted version.
